# The neurophysiological effect of NMDA-R antagonism of frontotemporal lobar degeneration is conditional on individual GABA concentration

**DOI:** 10.1038/s41398-022-02114-6

**Published:** 2022-08-27

**Authors:** Alistair Perry, Laura E. Hughes, Natalie Adams, Michelle Naessens, Alexander G. Murley, Matthew A. Rouse, Duncan Street, P. Simon Jones, Thomas E. Cope, Ece Kocagoncu, James B. Rowe

**Affiliations:** 1 MRC Cognition and Brain Sciences Unit, University of Cambridge, Cambridge, CB2 7EF UK; 2 Department of Clinical Neurosciences and Cambridge University Hospitals NHS Trust, University of Cambridge, Cambridge, CB2 0QQ UK

**Keywords:** Neuroscience, Predictive markers

## Abstract

There is a pressing need to accelerate therapeutic strategies against the syndromes caused by frontotemporal lobar degeneration, including symptomatic treatments. One approach is for experimental medicine, coupling neurophysiological studies of the mechanisms of disease with pharmacological interventions aimed at restoring neurochemical deficits. Here we consider the role of glutamatergic deficits and their potential as targets for treatment. We performed a double-blind placebo-controlled crossover pharmaco-magnetoencephalography study in 20 people with symptomatic frontotemporal lobar degeneration (10 behavioural variant frontotemporal dementia, 10 progressive supranuclear palsy) and 19 healthy age- and gender-matched controls. Both magnetoencephalography sessions recorded a roving auditory oddball paradigm: on placebo or following 10 mg memantine, an uncompetitive NMDA-receptor antagonist. Ultra-high-field magnetic resonance spectroscopy confirmed lower concentrations of GABA in the right inferior frontal gyrus of people with frontotemporal lobar degeneration. While memantine showed a subtle effect on early-auditory processing in patients, there was no significant main effect of memantine on the magnitude of the mismatch negativity (MMN) response in the right frontotemporal cortex in patients or controls. However, the change in the right auditory cortex MMN response to memantine (vs. placebo) in patients correlated with individuals’ prefrontal GABA concentration. There was no moderating effect of glutamate concentration or cortical atrophy. This proof-of-concept study demonstrates the potential for baseline dependency in the pharmacological restoration of neurotransmitter deficits to influence cognitive neurophysiology in neurodegenerative disease. With changes to multiple neurotransmitters in frontotemporal lobar degeneration, we suggest that individuals’ balance of excitation and inhibition may determine drug efficacy, with implications for drug selection and patient stratification in future clinical trials.

## Introduction

Frontotemporal lobar degeneration (FTLD) causes a diverse set of clinical syndromes, including behavioural variant frontotemporal dementia (bvFTD) and progressive supranuclear palsy (PSP) [1–3]. In addition to cell loss and atrophy [4, 5], the pathologies associated with bvFTD and PSP are associated with reductions in principal neurotransmitter systems that underlie cortical neurophysiology [6, 7], as measured by magnetoencephalography (MEG) [8–10]. Such neurotransmitter abnormalities are potentially remediable pharmacologically to restore physiology and thereby cognition. Pharmacological probes may also reveal mechanisms of disease, especially when coupled with simple tasks that patients can perform, such as roving oddball paradigms that evoke “mismatch negativity” responses (MMN) in frontotemporal networks [11, 12]. Mismatch negativity responses are generated in response to a surprising event that violates established patterns (e.g. a deviant tone with a high frequency after a sequence of standard tones of lower frequency). The mismatch response is derived from the difference in the neurophysiological responses between such deviant and standard events, typically maximal at 100–200 ms from stimulus onset [11, 13].

The expectation and response to sensory inputs depend on hierarchical information processing, in large-scale frontotemporal networks that are well suited to examine the impact of frontotemporal lobar degeneration. In oddball paradigms, standard regular stimuli establish strong predictions. When these predictions are not met (e.g. on deviant trials), an error signal is generated [13–15]. The MMN has been proposed to represent the precision of this error signal [16], which in turn determines the effect on future sensory expectations and perceptual inference [17–19]. Such predictions and error signals relay through the frontotemporal network, between the auditory and prefrontal cortex [20].

This signalling requires a critical interaction between Glutamatergic excitatory (E;) and GABAergic (y-aminobutyric acid) inhibitory (I;) activity (E/I balance) [15, 19, 21–23]. The former is dependent on N-methyl-D-aspartate receptor signalling (NMDA-R) [24], although the impact of NMDA-R treatments may be conditional on the GABA-ergic state [8, 25]. Preclinical and in vivo studies of disorders associated with FTLD have revealed reduced GABA and glutamate, particularly in prefrontal areas [7, 26–29]. These deficits contribute to neurophysiological abnormalities observed in both bvFTD and PSP, even in areas of minimal cortical atrophy. The physiological abnormalities include loss of beta-frequency desynchronisation and connectivity, reduced gamma oscillations and connectivity, and altered network integration [30–34]. Neurochemical and neurophysiological deficits correlate with cognitive and behavioural change [8, 26, 31], making them suitable intermediate phenotypes for experimental medicine studies.

Our focus on neurochemical rather than structural change is motivated by the potential for drug treatments. For example, memantine is a moderate affinity, uncompetitive NMDA-receptor antagonist. Licensed to treat Alzheimer’s disease [35, 36], it is generally well tolerated [37]. However, memantine was not clinically effective in two phase II studies of frontotemporal dementia [38, 39], although both were underpowered for clinical efficacy endpoints. It has also been a research tool to probe NMDA-R systems in neuropsychiatric disorders, including Schizophrenia [40]. Although there is patient heterogeneity in drug effects (c.f. Fig. 3 in ref. [40]), memantine can partially restore frontotemporal activity and connectivity, including auditory-based paradigms measuring early-level processing and MMN responses [40–42]. Here, we use memantine in the context of frontotemporal lobar degeneration.

Drug effects often show baseline dependency: too much or too little can both impair function. This means that group-wise tests may obscure significant effects of drugs that interact with individual differences [43, 44]. Measuring the concentration of the targeted neurotransmitter can reveal an effect of treatment in a subset of participants and suggest stratification for future trials [8, 43, 45]. For GABA and glutamate, proton magnetic resonance spectroscopy (^1^H-MRS) can quantify baseline neurochemical deficits. For example, the effect of GABA reuptake-inhibition on the MMN is conditional on baseline GABA concentration in the right inferior frontal gyrus [8]. Moreover, the balance between excitatory and inhibitory innervation suggests that memantine’s influence on neurocognitive deficits may also depend on GABA concentration [46–48]. Indeed, memantine [47, 49] and another NMDA-R antagonist ketamine [25, 50, 51], are posited to act upon excitatory inputs to inhibitory interneurons. This suggests a possible interaction between glutamate and GABA, leading to GABA-dependent moderation of the effect of memantine.

We aimed to determine the effect of memantine on frontotemporal neurophysiology in people with frontotemporal lobar degeneration and its relationship to baseline glutamate and GABA concentration. We consider bvFTD and PSP together despite molecular pathological differences because of their clinical, neurophysiological and neurochemical commonalities [1, 6, 9, 26]. First, we test the effect of memantine on magnetoencephalographic mismatch negativity responses in PSP and bvFTD versus controls. We focused upon the responses of regions within the frontotemporal network, given their involvement in oddball paradigms and frontotemporal lobar degeneration. Then, we test interactions between the drug effect and glutamate and GABA concentrations in the right inferior frontal cortex, exploiting the high signal-to-noise and spectral resolution of ^1^H-MRS at an ultra-high field (7T).

## Methods

### Subjects and pharmacological design

Twenty-four people with probable frontotemporal lobar degeneration (12 bvFTD, 12 PSP-Richardson’s syndrome) and 20 age-/sex-matched healthy adults undertook a randomised placebo-controlled double-blind crossover study (Table 1). Participants attended two magnetoencephalography sessions 2 weeks apart where they received either (1) 10 mg oral memantine or (2) placebo. Magnetoencephalography began 3 h after drug administration to coincide with peak blood levels [52]. Ten milligrams of memantine aligns with the clinically recommended starting dose in the UK [53].

**Table 1:** Demographic and neuropsychological information of study participants.

	CON	bvFTD/PSP	CON vs. bvFTD/PSP
	*M (SD)*		*p*-val (Bayes Factor)
*n*	19	20	
Sex	M14:F5	M18:2	n.s
Age	67.05 (4.72)	64.95 (8.49)	n.s (0.45)
*Neuropsychology*			
MMSE	29.63 (0.50)	26.9 (2.2)	*** (2598.18)
ACE-R			
Total	96.63 (2.54)	80.2 (11.03)	*** (48.52e+3)
Attention	17.89 (0.32)	16.95 (1.36)	** (8.09)
Memory	24.58 (1.47)	21 (4.22)	** (26.37)
Verbal Fluency	12.74 (1.52)	5.7 (3.4)	*** (1.07e+7)
Language	25.63 (0.83)	23.55 (2.19)	*** (65.86)
Visuospatial	15.79 (0.41)	13 (3.54)	** (21.43)
INECO			
Total	25.13 (2.23)	16.71 (5.4)	*** (26.52e+3)
FAB Total	17.16 (1.07)	13.32 (3.68)	*** (212.19)
Hayling			
Overall Scaled score	5.95 (0.85)	2.65 (2.01)	*** (10.89e+4)
Graded—naming total	25.47 (2.91)	17.68 (5.13)	*** (8.72e+e)
FRS Total (Logit)	4.23 (1.27)	−0.89 (2.26)	*** (1.16e+7)
CBI-R			
Total	5.67 (6.03)	68.32 (34.29)	*** (1.35e+6)
Memory and orientation	2.06 (2.16)	10.42 (7.23)	*** (492.72)
Everyday skills	0 (0)	8.74 (6.55)	n/a
Self-care	0 (0)	4.21 (4.76)	n/a
Abnormal behaviour	0.72 (1.02)	8.89 (8.18)	*** (134.55)
Mood	0.67 (1.14)	4.05 (3.01)	*** (269.54)
Beliefs	0 (0)	1.42 (1.8)	n/a
Eating habits	0.17 (0.38)	6.79 (4.96)	*** (5893.82)
Sleep	0.89 (1.23)	4.11 (2.72)	*** (331.85)
Stereotypic and motor	0.83 (1.15)	8.21 (5.72)	*** (2872.15)
Motivation	0.33 (0.69)	12.11 (5.52)	*** (4.72e+7)

n/a—variance in controls were equal to zero, violating assumption of equality of variances.

*p < 0.05.

**p < 0.01.

***p < 0.001, uncorrected.

*bvFTD* behavioural variant frontotemporal dementia, *CON* controls, *PSP* progressive supranuclear palsy,

*BF* Bayes Factor, Conventional thresholds for Bayes Factors represent substantial (>3), strong (>10) and very strong (>30) evidence in favour of alternate hypothesis.

*ACE-R* Addenbrooke's Cognitive Examination-Revised, *CBI-R* Cambridge Behavioural Inventory Revised, *FAB* frontal assessment battery, *FRS* Frontotemporal Dementia Rating Scale, *MMSE* mini-mental state exam.

Patients were recruited from tertiary referral centres with probable bvFTD, with or without parkinsonism [54] or probable PSP-Richardson’s syndrome (PSP-RS) [55], including those who had presented with “PSP-Frontal” phenotype [56]. Controls were recruited from the MRC Cognition and Brain Sciences Unit and NIHR Join Dementia Research. Participants had no history of significant neurological or psychiatric illness other than bvFTD/PSP. Written informed consent was acquired in accordance with the Declaration of Helsinki (1991). The study was approved by the local ethics committee and exempted from Clinical Trials status by the UK Medicines and Healthcare products Regulatory Agency. The International Standard Randomised Controlled Trial Number is 10616794. One control was excluded because they did not complete both a placebo and drug magnetoencephalography session. After quality control review, four patients were excluded from the analysis because they (i) lacked a N70 in the auditory cortex (*n* = 1), (ii) had fewer than half the average number of trials after artifact rejection (*n* = 1) or (iii) was unable to complete two magnetoencephalography sessions (*n* = 2).

The neuropsychological assessment included the revised Addenbrookes Cognitive Examination (ACE-R) [57], Frontal Assessment Battery (FAB) [58], Graded Naming Test [59], INECO Frontal Screening Test [60] and Hayling test [61]. A close informant completed the revised Cambridge Behavioural Inventory (CBI-R) [62] and Frontotemporal Dementia Rating Scale (FRS) [63]. To derive each patient's disinhibition phenotype score, we calculated a composite score from the following CBI-R subscales [31, 64]: abnormal behaviour, stereotypic movement and behaviour and eating.

### Magnetoencephalography, preprocessing and source localisation

Magnetoencephalography was recorded during a passive roving auditory paradigm [20, 23] (Supplementary Information 1.1). In brief, participants heard a series of repeated tones *(rep*_*n*_) at a given frequency (400–800 Hz, 75 ms), with stimulus-onset-asynchrony 500 ms. The tone frequency changed pseudorandomly after 3–10 repetitions (approximate Poisson distribution). The first new tone is classed as deviant (*dev*). The paradigm was performed with eyes-open in three blocks of 5 min, while participants watched a silent movie. After trials rejection, participants averaged 1577 (SD = 109) stimuli per session.

Magnetoencephalography used a magnetically shielded room (IMEDCO) and the Elekta VectorView system (Elekta Neuromag, Helsinki), with 306-channel recordings at 102 spatial locations (planar gradiometer pair and magnetometer at each site), sampled at 1000 Hz. Vertical and horizontal Electro-occulography (EOG) indicated eye movements and 5 head position indicator coils tracked head position. A 3D digitizer (Fastrak Polhemus Inc., Colchester, VA) was used to record nasion and pre-auricular fiducial point positions and >60 scalp surface points.

Preprocessing used SPM12 (v7771), FieldTrip [65] and OSL (https://github.com/OHBA-analysis/osl-core) software in Matlab (2019a) (pipeline available at https://github.com/AlistairPerry/FTLDMEGMEM). First, MaxFilter (v2.2.12, Elekta Neuromag) was used to interpolate bad channels, remove external noisy signals (using signal source separation) and correct for head motion. The data were next downsampled (500 Hz), band-pass filtered (0.1–125 Hz) and notch filtered (removing frequencies between 45–55 Hz and 95–105 Hz) [66]. Bad channels (using *osl_detect_artefacts*) and eye-movement-related artifacts were removed with independent component analysis. Data were epoched between −100 to 400 ms relative to tone onsets, with bad trials removed (*osl_detect_artefacts*) and then averaged. A 125 Hz low-pass filter removed high frequencies. Baseline correction was applied −100 to 0 ms (Supplementary Information 1.2).

Magnetoencephalography signals were source localised using all channels, in SPM12 using a single shell cortical mesh, estimated from individual T1-weighted images (co-registered using fiducial and head points). A canonical template was used for three people who did not have MRI of sufficient quality (one patient, two controls). The evoked source signals were estimated for each trial type using COH inversion (sLORETA [67]). We extracted waveforms from literature-specified MNI coordinates of cortical MMN sources [20, 23]. We focus on the right-hemisphere regions given right lateralised MR Spectroscopy: auditory cortex (AUD; [46,−14,8]), superior temporal gyrus (STG; [59,−25,8]), and inferior frontal gyrus (IFG; [46,20,8]). The local peak was identified within a 7 mm radius and extracted to form an average pseudo-local field potential (LFP) response. We also estimated the average event-related fields (ERFs) from all gradiometers, and frontal and temporal gradiometers (Supplementary Fig. 4B), which were baseline corrected and smoothed (moving average 20 time-points).

We focus on the deviant trial (*dev*) and third repetition (*rep3*). For both sensor and source data, we calculated a difference waveform from the *dev* and *rep3* (*rep3*-*dev*) responses, to derive the mismatch response. The mean MMN was calculated from the mean mismatch response between 125 and 175 ms post-stimulus presentation, based on independent data [11]. This was our chosen contrast and window, as prior MMN studies show electro-/magnetoencephalography peaks within this window, and a response plateau emerging by the third repetition [16, 68]. We corroborated this in an independent age-matched control cohort (mean age = 66.42) [23] (Supplementary Information 1.3, Supplementary Fig. 1). This early plateau indexes short-term plasticity and learning.

### MR imaging and spectroscopy

Participants completed a T1-weighted MP2RAGE structural scan at 7 T on a Siemens TERRA system (Siemens Healthineers, Erlangen, Germany) with 32 channel headcoil. Acquisition parameters were: 0.75 mm isotropic voxels, TE = 1.99 ms, TR = 4300 ms, inversion times = 840 ms/2370 ms) (Supplementary Information 1.4). Two patients were unsuitable for 7 T and underwent 3 T scanning (Siemens PRIMSA MPRAGE; 1.1 mm isotropic voxels TE = 2.9 ms, TR = 2000 ms). Two controls were unsuitable for MRI.

**Fig. 1:**
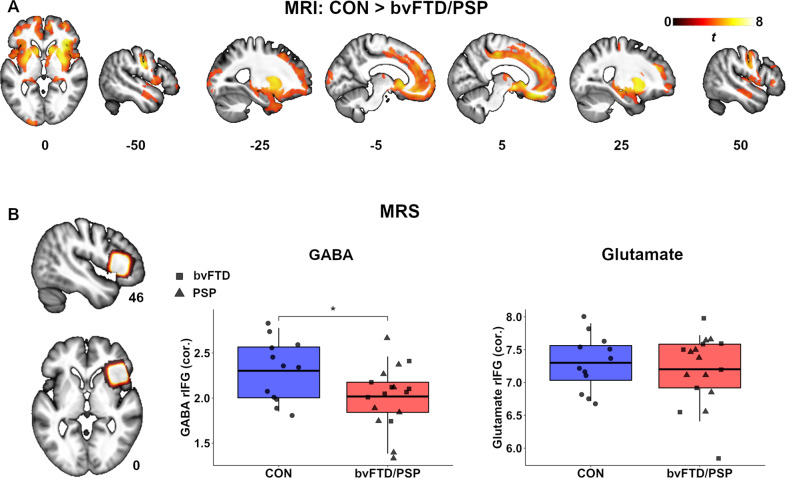
Neuroanatomical and neurochemical differences across controls and persons with bvFTD/PSP. **A** Group-wise voxel-based morphometry comparisons between controls and patients (cluster-level, *p* < .05, FWE-corrected; height-threshold, *p* < 0.001, uncorrected). Unthresholded SPM maps are available at https://neurovault.org/collections/12279. **B** MRS concentrations of GABA (left panel) and Glutamate (right) in the right inferior frontal gyrus for both controls and patients (bvFTD, squares; PSP, triangles) corrected for age, sex and partial-volume information (grey and white matter for glutamate, grey matter for GABA). Heat voxel image (far left panel) represents sum of all participants MRS voxel placement in the frontal cortex. Anatomical images are the mean brain extracted structural image across all controls and patients. *p < .05, uncorrected. bvFTD behavioural variant frontotemporal dementia, PSP progressive supranuclear palsy.

To determine whether magnetoencephalography responses to memantine are dependent on GABA and/or glutamate concentrations, we used ultra-high field 7 T MR spectroscopy (MRS, Siemens TERRA) [26] (Supplementary Information 1.5). We focus on the association between MRS and magnetoencephalography in patients based on a disease-specific variance in neurochemistry. Three patients had incomplete scans or substantial movement artifacts. MR spectra were acquired from voxels of interest (2 × 2 × 2 cm^3^) in the right inferior frontal gyrus [26] (for voxel placement see Fig. 1B); and the occipital cortex as a control region. Spectra were acquired using a short-echo semi-LASER sequence [69, 70] (repetition time/echo time = 5000/26 ms, 64 repetitions). Eddy-current effects and frequency and phase shifts were corrected by using MRspa (University of Minnesota, www.cmrr.umn.edu/downloads/mrspa).

Glutamate and GABA were quantified using LCModel (Version 6.2–3) [71]. For partial-volume correction, SPM12 was used for segmentation and estimation of tissue-type probabilities in each voxel. Grey matter volume was used to correct for GABA, and grey and white matter volume for glutamate. A generalised linear model controlled for the effect of age, sex and partial-volume information [26]. Non-corrected MRS values are also presented.

To test whether atrophy in the right inferior frontal gyrus could also account for differences in the MEG responses to drugs, we estimated individual grey matter volume. T1 images were bias regularised (threshold of 0.0001), tissue-segmented (SPM12 v7771), normalised to MNI space via differomorphic registration [72], and spatially smoothed (for VBM only) (8 mm FWHM) (Supplementary Information 1.6). Grey matter volume (GMV) in the inferior frontal gyrus was calculated from a right-hemisphere anatomical mask of Brodmann areas 48, 49 and the frontal operculum (OP8) (github.com/inm7/jubrain-anatomy-toolbox) [73], overlapping the magnetoencephalography source region (Fig. 5A; Supplementary Information 1.7; https://neurovault.org/images/776918). We also investigated grey matter atrophy elsewhere using voxel-based morphometry (VBM) [74] (Supplementary Information 1.8).

### Statistical analysis

Given the shared phenotypic deficits between bvFTD and PSP individuals [1, 8], principal analyses were conducted by pooling over patient groups. In auxiliary analyses, disease subgroups (PSP vs. bvFTD) were compared separately. This involved a one-way ANOVA for group differences on placebo and 2 × 3 design for subgroup × drug interactions. For clinical and neuropsychological descriptives, we present bvFTD and PSP subgroups separately (with Tukey correction).

### Group differences in MMN responses on placebo and responses to Memantine

Independent *t*-tests (two-tailed) assessed group differences in mean MMN on placebo for sensor and source region waveforms. Factorial 2 × 2 ANOVAs assessed differential group responses to memantine (group × drug interaction) in source regions, with drug session and group the within- and- between-subject factors, respectively.

Pearson correlations tested the association between patients' GABA and glutamate concentrations with change in mean MMN responses to the drug, relative to placebo. Stepwise linear regression was used to determine whether MRS associations with drug-dependent responses were confounded by other factors, including disease subgroup, age, and placebo MMN responsivity.

Descriptive frequentist statistics were performed in MATLAB 2019a, and their corresponding Bayesian analyses were conducted in JASP. In contrast to frequentist *t*-tests or analysis of variance, which may fail to reject the null hypothesis but cannot support it, the Bayesian tests can provide evidence for the null hypothesis (e.g. of no group effect or no drug effect). For sensor and source analyses involving multiple regions, Bonferroni correction (α = 0.0167 [0.05 / three source regions]) was used. For Bayesian tests, Bayes Factors (BF_10_) represented moderate (>3) or strong (>10) evidence against the null hypotheses, while <0.33 (moderate) and <0.10 defined (strong) evidence in favour of the null. For Bayesian ANOVAs the Bayes Inclusion Factor was calculated.

Power and sample size calculations are approximate, given the novelty of the study. Twenty individuals per group are in line with previous neurophysiological studies of crossover drug-placebo effects of bvFTD and PSP [8, 75], and memantine in schizophrenia [41]. Using a different mismatch paradigm, Cope et al. [76] and Hughes et al. [9] reported 30–40% reductions in amplitude with bvFTD (Cohen’s *d* > 1.6). In this context, for frequentists tests, *N* = 20 provides >80% power with alpha 0.05 to detect a restorative crossover drug intervention *versus* placebo with effect size *d* ≥ 0.6 [77] (noting the smaller *d* = 0.3 reported by [41] in control participants); and >80% power with alpha 0.05 to detect a correlation *r* ≥ 0.5 with GABA. For Bayesian inference, the concepts of power, type I and II error are not directly applicable. An insufficient sample for the size of the effect (i.e. inadequate precision in the data) would be reflected in an indeterminate Bayes Factor (BF), 0.33 < BF < 3.

## Results

Patients and controls did not differ in age or sex (Table 1). As expected, both bvFTD and PSP patients were impaired in the INECO, FAB, Graded Naming test, FRS, Hayling and selected subscales of the ACE-R and CBI-R. Compared to PSP, bvFTD patients performed worse on the Hayling (A + B error score) and selected CBI-R subscales but did not differ in terms of MMSE, Hayling (overall scaled score), ACE-R subscales, FRS or FAB (Supplementary Table 1).

While the paradigm presented the same number of trials across control and bvFTD/PSP individuals, the number of trials removed differed between the groups (in line with ref. [8]) due to a higher rate of artifacts in the patient group (e.g. eye blinks, occasional movements or swallowing) (Supplementary Table 2). The MRS water line width did not differ across groups (Supplementary Table 2). GABA Cramer-Rao lower bounds (reflecting uncertainty in measurements) were higher in patients, but this did not survive Bonferroni correction.

Patients had bilateral atrophy in frontal, temporal, thalamic, striatal, and left occipital regions (Fig. 1A, Supplementary Table 3; cluster-level, *p* < .05, FWE-corrected; height-threshold, *p* < 0.001, uncorrected); with reduced grey matter in bvFTD relative to controls in medial frontal, medial temporal, striatal and insular areas. PSP demonstrated reduced grey matter in frontal, temporal, striatal, insular and hippocampal regions (Supplementary Fig. 2). bvFTD and PSP groups did not differ significantly.

Partial-volume corrected GABA concentration in the right inferior frontal gyrus was reduced in patients, but weakly at the group level (bvFTD and PSP combined; *df* = 27, *p*_unc_ = 0.036, BF_10_ = 2.01). Glutamate concentration did not differ (*p*_unc_ = 0.60, BF_10_ = 0.39; Fig. 1B). See Supplementary Table 4 for each group separately. Uncorrected concentrations were reduced in patients for both GABA (*p*_unc_ < 0.001, BF_10_ = 31.66) and Glutamate (*p*_unc_ < 0.001, BF_10_ = 67.13).

Corrected GABA values in patients were not associated with cognitive screening scores (i.e. ACE-R, FAB) or clinical function markers (i.e. FRS), or the behavioural disinhibition score. Corrected glutamate concentrations were strongly associated with Frontal Assessment Battery (FAB) (*p*_unc_ = 0.004) and weakly with ACE-R (*p*_unc_ = 0.042) (Supplementary Fig. 3).

### Group differences in physiology

Averaged across all sensors, clear mismatch responses were observed for both groups, with average peak negative deflection (*rep3*-*dev*) at ~150 ms (Supplementary Fig. 4A). The groups did not differ in mean MMN (125–175 ms) at any of the regional sensor groups (*df* = 37, *p*_unc_ > 0.08, BF_10_ = 0.65–1.07; Supplementary Fig. 4C). The lack of a group difference was also observed in comparison to the independent control cohort (*df* = 37, *p*_unc_ > 0.09, BF_10_ = 0.5–0.1) (Supplementary Fig. 5).

**Fig. 2:**
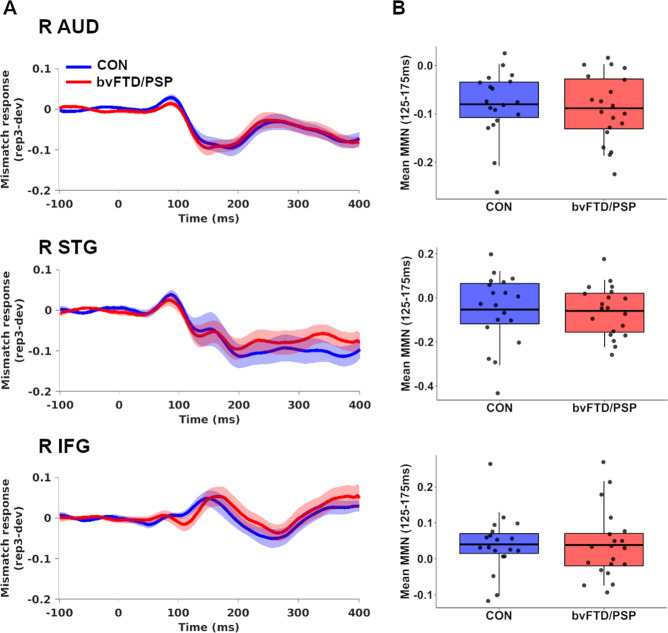
Mismatch responses of source regions across controls and bvFTD/PSP patients on placebo. **A** Group average mismatch responses across peri-stimulus window for controls (blue line) and bvFTD/PSP (red), derived from each individual by the difference of the *rep3* and *dev* waveforms. Thick lines and shading represent group average and its standard error at each time-point, respectively. **B** Mean MMN responses (average of mismatch waveform between 125 and 175 ms) in controls and patients, with middle line indicating the group mean. Boxes represent interquartile range of 25% and 75% percentile, with whiskers indicating 95% probability density. R IFG right inferior frontal gyrus, R STG right superior temporal gyrus, R AUD right auditory cortex, bvFTD behavioural variant frontotemporal dementia, PSP progressive supranuclear palsy.

For all source regions (see Supplementary Fig. 6 for single-condition waveforms) we also observe no group differences in mean MMN between all patients and controls (*df* = 37, *p*_unc_ > 0.71, BF_10_ = 0.31–0.33; Fig. 2). Levene’s tests confirmed that across all source regions, the variance in mean MMN was similar across control and patient groups (*p*_unc_ > 0.22). For comparisons across three groups (controls, bvFTD and PSP) there was a group effect in the right inferior frontal gyrus (*df* = (2,36), *p*_unc_ = 0.01, BF_incl_ = 4.51, Supplementary Fig. 7A, Supplementary Table 5); MMN responses were reduced in bvFTD compared to PSP (*df* = 18, *p*_tukey_ = 0.007, BF_10_ = 10.42).

**Fig. 3:**
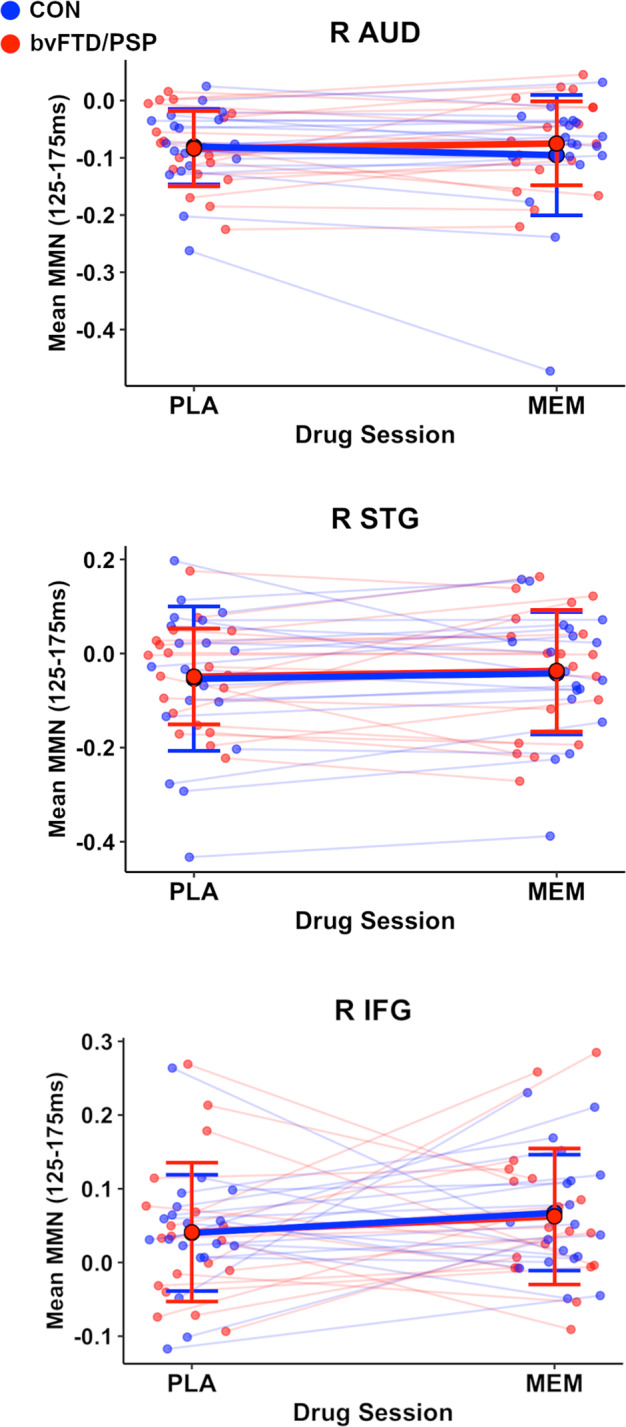
Group responses to memantine across controls and persons with PSP and bvFTD. Mean MMN response across placebo (PLA) and memantine (MEM) drug sessions for controls (blue circles and lines) and FTLD (red). Mean group responses at each drug session are indicated by bold circles, with error bars representing 95% confidence intervals. Group average change in mean MMN across drug session indicated by solid lines, with each participants slope illustrated by opaque lines. R IFG right inferior frontal gyrus, R STG right superior temporal gyrus, R AUD right auditory cortex, bvFTD behavioural variant frontotemporal dementia, PSP progressive supranuclear palsy.

We next tested whether memantine has a differential influence on source mismatch responses between controls and patients, relative to placebo. Group-wise analysis revealed that no region exhibited a group × drug interaction (*df* = (1,37), *p*_unc_ > 0.16, BF_incl_ = 0.28–0.75; Fig. 3), even if the patient subgroups were differentiated (*df* = (2,36), *p*_unc_ > 0.17, BF_incl_ = 0.24–0.69, Supplementary Fig. 8).

While we find no evidence for a differential group effect of memantine on the planned time-averaged MMN (125–175 ms), we explored for each individual the difference in the mismatch response across drug and placebo, and compared groups in this drug difference at each time-point (i.e. difference of differences). Memantine had a weakly differential group effect on early responses (70–134 ms) in the right auditory cortex (*p*_unc_ < 0.05 uncorrected-threshold only; grey markers, Supplementary Fig 9A). Paired *t*-tests indicate this effect is driven by an increased auditory mismatch response in patients on the drug (dotted red line) relative to placebo (dashed line), occurring in early-sensory components, 80–124 ms (Supplementary Fig. 9B).

### GABA and glutamate influences on the response to Memantine

**Fig. 4:**
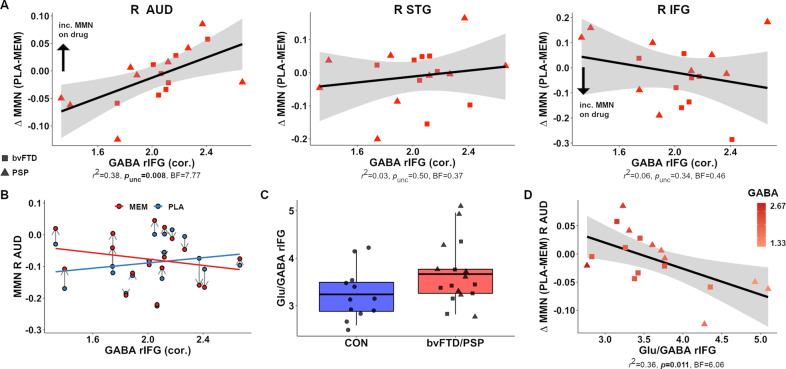
GABA influences on the response to memantine in patients across source regions. **A** Association between corrected GABA concentrations in the R IFG and change in MMN to memantine (vs. placebo) across bvFTD (squares) and PSP patients (triangles). **B** For the right auditory cortex, mean MMN as a function of drug session (Drug, red line and circles; Placebo, blue) and GABA concentration. **C** Glu/GABA ratios in controls and bvFTD/PSP, with middle line indicating group mean. Boxes represent interquartile range of 25% and 75% percentile, and whiskers the 95% probability density. **D** Association between the ratio of Glutamate and GABA (Glu/GABA) and change in MMN to memantine (vs. placebo) in the right auditory cortex. Data points are coloured according to baseline GABA levels. bvFTD behavioural variant frontotemporal dementia, PSP progressive supranuclear palsy, R IFG right inferior frontal gyrus, R STG right superior temporal gyrus, R AUD right auditory cortex. PLA placebo, MEM memantine. GABA and Glutamate concentrations corrected for age, sex and partial-volume information.

We tested baseline dependency of drug effects. Individual GABA concentration moderated the patients’ MMN response (*dev-rep3*) to memantine in the right auditory cortex (Fig. 4B, far left panel), with moderate-to-strong evidence in favour of this association (*df* = 15, *p*_*unc*_ = 0.008, BF_10_ = 7.77): Patients with higher GABA concentration showed larger MMN responses (i.e. more negative MMN) on memantine relative to their placebo session (Fig. 4B). Robust regression confirmed this relationship is robust to potential influencing observations (*b* = 0.093, *p* = 0.009). There was no moderating effect of glutamate on drug-dependent mismatch responses in any region (*p*_unc_ > 0.41; Supplementary Fig. 10). This association, and the non-significant relationship with glutamate, remained if using MRS concentration without partial-volume correction (Supplementary Table 6).

Stepwise regression with additional predictors, including disease subgroup, age, and baseline (placebo) MMN responses, confirmed GABA concentration as the best predictor of patients’ MMN (*rep3-dev*) response to memantine *(F* = 9.38, *p* = 0.0079). In previous work, memantine’s effect (20 mg) in schizophrenic patients was moderated by age [41]. However, the relationship between prefrontal GABA and MMN change to memantine in our study is not moderated by age (*p*_AgexGABA_ = 0.42) (Supplementary Fig. 11).

The ratio between glutamate and GABA (Glu/GABA) concentrations has been used as a proxy of cortical excitatory/inhibitory balance [48, 78]. Glu/GABA ratios in patients were negatively associated with the mean MMN change on memantine (vs. placebo) in the auditory cortex (Fig. 4D). This was not observed in controls (*df* = 12, *r*^2^ = <0.01, *p* = 0.90, BF_10_ = 0.36), although an interaction between group and Glu/GABA ratio on the change in MMN was non-significant (ANCOVA; *df* = (1,25), *p* = 0.23). While a comparison of Glu/GABA ratios to controls indicates no difference (*p* = 0.078, BF_10_ = 1.20; Fig. 4C), it does suggest patients with greater MMN responses to drugs are those with relatively preserved Glu/GABA concentrations.

**Fig. 5:**
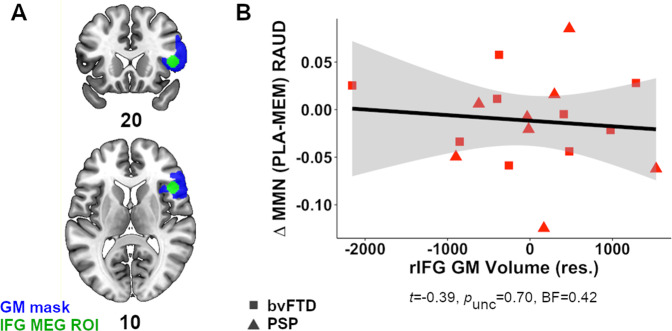
Association between prefrontal cortical atrophy and changes in MMN response to memantine in auditory cortex. **A** Anatomical mask (blue areas) representing right inferior frontal gyrus (rIFG) region used for calculating GM volume, as visualised from axial and coronal views. Mask is available at https://neurovault.org/images/776918. Green sphere (7 mm radius) indicates magnetoencephalography (MEG) source region-of-interest (ROI) used in MMN analysis. **B** Association between residual GM volume (adjusted for age and total intracranial volume) in rIFG and change in MMN to memantine (vs. placebo) (PLA-MEM) across bvFTD (squares) and PSP patients (triangles). bvFTD behavioural variant frontotemporal dementia, PSP progressive supranuclear palsy;, R AUD right auditory cortex, R IFG right inferior frontal gyrus.

Atrophy of the prefrontal cortex (controlling for age and total intracranial volume) neither moderate patients’ magnetoencephalographic response to memantine in the auditory cortex (*df* = 15, *p*_unc_ = 0.70, BF_10_ = 0.42; Fig. 5B), nor did cognitive (ACE-R, *df* = 18, *p*_unc_ = 0.28, BF_10_ = 0.48; FAB, *df* = 17, *p*_unc_ = 0.49, BF_10_ = 0.35) or clinical severity (FRS, *df* = 17, *p*_unc_ = 0.68, BF_10_ = 0.31). Occipital GABA concentration was not associated with drug-dependent MMN changes in auditory cortex (*p* = 0.21, BF_10_ = 0.62).

## Discussion

The principal finding is that in people with syndromes associated with frontotemporal lobar degeneration, the neurophysiological responses to the NMDA-R antagonist memantine are conditional on individual GABA concentration. Patients with relatively preserved GABA concentrations have greater enhancement of the mismatch responses (i.e. more negative MMN to memantine vs. placebo). This effect was neither explained by regional atrophy, nor the phenotypic factors of age or disease subgroup. We suggest that in the context of future experimental medicine studies, magnetic resonance spectroscopic quantification of heterogeneity might be useful for stratification, according to multiple neurotransmitter deficits. Otherwise, within-group neurochemical heterogeneity is liable to reduce sensitivity to treatment effects and increase type II error in clinical trials.

The selectivity of the auditory cortex MMN changes to memantine is not unexpected. For example, in Schizophrenia, there is both NMDA-R dysfunction and consistent abnormalities in auditory MMN [79]. Auditory regions are sensitive to memantine in both Schizophrenia and controls with drug modulation of neural responses [40–42]. However, frontotemporal lobar degeneration, with bvFTD and PSP, is also associated with prefrontal cortical atrophy and GABA-ergic deficits. Neurophysiological changes can occur prior to atrophy or in the absence of atrophy. This is in part because of the loss of synapses [80, 81] and reductions in critical neurotransmitters [6] in bvFTD/PSP [32, 33, 82, 83] and other neurodegenerative disorders [84–86]. Magnetoencephalography, or electroencephalography, may therefore provide sensitive markers of disease progression and drug response. In this study, there was a group-wise reduction in GABA concentrations in patients [8, 26], as expected from post-mortem data [87], but the distribution was wide. This variation in GABA, not atrophy, correlated with the effect of memantine on the cortical MMN response.

The drug response in auditory cortex was conditional on frontal GABA status, two areas that span the hierarchical neurocognitive network for prediction and response in MMN tasks [11, 88]. The auditory cortex is relatively spared by the direct neuropathology of bvFTD and PSP, but within the hierarchical network for prediction and error signalling [89, 90], its response is conditional on backward projections from the association cortex. A general feature of sensory processing hierarchies is that feedback and feedforward connections are shaped by laminar specificity in cortical units: feedforward connections project principally from superficial pyramidal cells, while feedback connections arise particularly from deep pyramidal cells and terminate on superficial layers at lower-level regions such as the auditory cortex [15, 89, 90]. Prefrontal GABA regulates the precision of the frontotemporal predictions and the deep cortical generators of back-projecting beta-oscillations [8, 23]. Indeed, beta power and beta-connectivity are reduced in PSP and several syndromes of frontotemporal dementia [8, 9, 31, 91]. The effect of memantine on mismatch response (MMN) generation, mainly from superficial cortical layers of lower levels of the sensory hierarchy, is thereby moderated by prefrontal GABA. In other words, the mismatch between incoming deviant auditory signals with the predicted standard tone is larger on memantine in the context of (near) normal prefrontal GABA.

An alternative hypothesis is that GABA measurements in the inferior frontal gyrus index widespread GABAergic deficits. However, drug-dependent responses in the auditory cortex are independent of GABA concentrations in the occipital cortex, and both the occipital lobe and auditory cortex are relatively spared by direct neuropathology.

The MMN differs across many neurological and psychiatric disorders, including Schizophrenia [79, 92], Alzheimer’s disease [93] and neurodevelopmental conditions [94]. The MMN provides a robust marker of frontotemporal functioning and is well tolerated in clinical populations. Our lack of a significant group difference in MMN response is unexpected from previous reports of bvFTD [8–10]. The difference might arise from variations in MMN paradigms or heterogeneity of the disease, including variance in GABA concentration, atrophy, and syndrome. Indeed, considerable variation has been reported across Schizophrenic patients [92] across studies using the MMN. Patient heterogeneity may undermine power unless the causes of heterogeneity are factors in the analyses. Future studies with larger samples are required to test whether the magnitude of MMN response scales with clinical or cognitive impairment or neurochemical variance. We also note the heterogeneity in atrophy across PSP subtypes in a larger cohort [95]. Our planned comparisons pooled PSP and bvFTD patients because of their commonalities in physiology and phenotype noted in other deep phenotyping studies [1, 55], despite the clear differences in underlying molecular pathology. Consistent with the ‘frontal’ cognitive deficits in PSP, the majority of cognitive tests were similarly affected by both groups. Although the auditory MMN did not differ between groups, the supplementary analyses suggest prefrontal MMN differences with a particularly blunted response in the frontal cortex on placebo in bvFTD. The subgroup sample sizes (*n* = 10 in each group) could be considered relatively small, but Bayesian tests indicate strong evidence in favour of a group difference. The absence of a main case-control effect should be interpreted in the light of interactions, such as with the neurochemical variance to which we turn next.

While memantine is an NMDA-R antagonist targeting glutamatergic functioning, the response to the drug was conditional on GABA rather than glutamate concentration. There are several possible interpretations. The first is that the MRS-measured glutamate is not only exclusively neuronal and available for neurotransmission but also astrocytic as part of glutamate-glutamine cycling [96]. Second, an interaction between glutamatergic and GABAergic systems reflects a delicate balance between excitatory (E) and inhibitory (I) control of the firing of neuronal ensembles. Neurophysiological proxies of E/I functioning have found changes to this balance with ageing [97] and neurodegeneration [98]. In this study, we tested the ratio of glutamate/GABA concentrations as a proxy of an individual’s E/I balance [48] and found that patients with relatively normal glutamate to GABA ratio show increased mismatch responses to memantine. Importantly, memantine and another NMDA-R antagonist, ketamine, increase pyramidal output activity through their excitatory inputs to GABA interneurons [47, 50, 51]. We speculate that this interaction between prefrontal GABA and memantine promotes coordinated pyramidal firing in response to deviant tones in the oddball paradigm. Such an effect of memantine on the E/I balance has been proposed in Schizophrenia via influences on oscillatory dynamics [40, 46] according to the ratio of glutamine to glutamate [99].

Note that memantine’s effects were moderated by a neurochemical (GABA) that is not its primary target (glutamate receptors); and its concentration in a region (i.e. prefrontal cortex) that is connected to but non-overlapping with the generator of the dependent measure (i.e. auditory cortex). This is not unique in clinical neuroscience: consider, for example, the interactions between opioid or serotonergic treatment effects on dopamine status [100, 101]. Subject to replication, this indirect approach has implications for experimental studies, in which stratification may need to be considered on basis of interactions between neurotransmitter systems or between connected regions. We propose that the design of future experimental studies may benefit from the quantification of multiple neurotransmitter systems, including neurochemicals changed by disease and those targeted by drugs. Here, GABA concentrations were an anticipated influence (along with glutamate) on individual differences in patient responses to memantine, given that baseline levels moderate GABAergic modulation [8] and are associated with variation in behaviour [26]. Moreover, the effect of other NMDA-R antagonists (i.e. ketamine) on GABA populations is well established [47, 50, 51], as are the dysfunctional interactions between GABA-and-glutamatergic cells in other NMDA-R disorders (i.e. Schizophrenia) [102]. Unfortunately, spectroscopy of the auditory cortex was not available, and the frontal lobe was prioritised. With respect to the drug responses of brain regions investigated, we recommend the regions to be measured include those that are functionally probed by the experimental design. Our choice of three frontotemporal nodes was relatively straightforward in the context of the mismatch negativity paradigm [20, 88]. Indeed, cortical sensory areas in the mismatch network have previously been implicated in bvFTD [9, 10], Schizophrenia [9, 10] and are sensitive to modulation by other drugs used to treat dementia [16]. A potential approach for a priori selection of regions for outcome variables is to use the information on neurotransmitter receptor distributions across the cortex [103], derived, for example, from PET receptor maps [104] or a transcriptomic atlas. Regions preferentially targeted by the drug are most likely to show drug-induced changes and perhaps even exhibit within-patient heterogeneity.

The selection of multiple interacting neurotransmitter systems and brain regions used in stratification raises the potential challenge of too many “researcher degrees of freedom” and multiple comparisons. Our findings would benefit from independent replication and consideration of data-reduction methods [105, 106] or prediction algorithms (i.e. lasso) for identifying or stratifying (clusters of) drug-sensitive individuals.

Another consideration for future studies is the degree to which baseline pathology moderates a drug response. Our findings can be interpreted as relatively greater pathology (with less GABA) leading to decreased sensitivity to memantine. This is, therefore, not a simple restoration of function in those with more severe baseline deficits [43]. While it may be easier to show a drug effect than prove its absence, the patients’ disease severity and heterogeneity will affect the result of analyses of a drug’s group-wise main effect.

There are limitations and caveats to this study in pharmacology, diagnosis and analysis. First, we note that memantine does not have clinical trials evidence for efficacy and has been subject to successful (but negative) small phase II trials. We do not advocate its clinical use in either PSP or bvFTD. This study was not a clinical trial. Rather, we used the drug as a well-tolerated psychopharmacological probe of neural systems. We found no differential effect of memantine 10 mg on the evoked MMN between groups. In both healthy controls and Schizophrenia, a higher 20 mg memantine dose changed the mismatch response [41], whereas 10 mg produced no group-wise effect [40, 41]. It’s important to note the age of control (mean = 27.51) and schizophrenic participants (36.44) [41] is considerably younger than those in the current study, and data were acquired with electrophysiology (EEG) rather than MEG, and employed a different mismatch paradigm with the spatiotemporal calculation of MMN. This calls for caution in a direct dose comparison across the studies. Moreover, the memantine effect in schizophrenia was moderated by age [40]. Our 10 mg dose was chosen to align with the clinically recommended starting dose in the UK [53], but future studies may consider higher dosages that balance tolerability and physiologically efficacy. Although on further inspection, we do find that memantine appears to subtly increase early-auditory mismatch responses in patients. Interestingly, this effect is again unique to the auditory cortex and is consistent with multiple studies revealing memantine’s influence (at 20 mg) on early-auditory processing in schizophrenic patients [40, 41]. These effects, however, do not survive correction for multiple comparisons and thus, we cannot make strong conclusions about the drug’s effectiveness. Our current null findings (for the MMN) do not necessarily imply that the MMN is not a sensitive marker of disease or drug response—the patient heterogeneity, relatively small sample size, and paradigm may together work to yield non-significant group differences on placebo. The effects of memantine may not be strong enough or consistent enough to produce an overall group effect on frontotemporal networks. Despite the current result with memantine, previous evidence of the modulation of frontotemporal networks with GABA agonists in FTLD [8] highlights the potential of the MMN as a marker of drug effects.

While our study focused on disease-modifying treatments of neurochemical deficits, non-invasive brain stimulation has also emerged as a promising technique for elucidating and restoring abnormal physiological inhibition/excitation arising from neurotransmitter abnormalities. Single sessions of either transcranial direct current stimulation (tDCS) or transcranial magnetic stimulation (TMS) reveal reductions in both intracortical inhibition (GABAergic) and excitation (gluamatergic) in FTLD syndromes [107]. Moreover, individual differences in these neurophysiological markers distinguish FTLD from other neurodegenerative disorders (i.e. Alzheimer’s) [107] and have been associated with positive (i.e. disinhibition, aggressive behaviour) and negative symptoms (i.e. apathy, aspontaneity) [108]. We similarly found glutamate concentrations to correlate with frontal lobe functioning (i.e. FAB). Repeated tDCS over 2 weeks can lead to a restoration of both glutamatergic and GABAergic functioning in bvFTD and PPA, together with improvements in cognitive and clinical functioning [109]. These findings suggest that pharmacological intervention and non-invasive brain stimulation might be used in combination to modify neurotransmitter abnormalities for therapeutic benefit in the context of FTLD.

Our patient diagnoses were clinical, not genetic, or neuropathological. Clinicopathological correlations are very high for both PSP and bvFTD, although we cannot distinguish the Tau *versus* TDP43 pathology as the basis of the bvFTD cases. In the main analyses, we pooled PSP and bvFTD groups despite the clear differences in underlying molecular pathology, not for power considerations but because of the commonalities in physiology and phenotype noted in deep phenotyping studies [1, 55]. Consistent with the literature on ‘frontal’ cognitive deficits in PSP, patients with PSP and bvFTD were similarly impaired on many of the same cognitive tests, with limited selectivity of deficits in bvFTD. Statistical power and precision are key considerations, especially with *n* = 20 per group. We used a crossover design that increases power relative to parallel groups designs for heterogeneous populations and leveraged Bayesian inference to consider the evidence in favour of the null hypothesis, as well as alternate hypotheses. For our principal finding, despite modest numbers, there was sufficient precision to draw inferences, with positive or moderate-to-strong evidence for the association between GABA concentration and change in MMN responses on memantine (BF_10_ > 7). However, the lack of an overall group effect of memantine, and the association between drug effect and GABA concentration, requires replication in an independent study. Only a subgroup of controls completed MRS (*n* = 12), so we did not attempt correlations with neurotransmitter levels within the control group. For MRS-based analysis of patient effects, the ratio of glutamate/GABA concentrations as a proxy of an individual’s E/I balance has recently been challenged [78] in favour of other neurophysiological measures such as the 1/f aperiodic slopes [46], but the resolution of that debate is beyond the scope of this study.

In conclusion, we have probed neurocognitive systems in two disorders associated with frontotemporal lobar degeneration, combining memantine pharmacological challenge with magnetoencephalography and ultra-high field spectroscopy. Patients’ neurophysiological responses to memantine were proportionate to GABA concentration. It may be possible to de-risk the transition from experimental medicine to clinical trials of heterogeneous populations using neurophysiological outcomes and stratification by spectroscopy.

## Supplementary information

Supplementary Material I

Supplementary Table 1

## Data Availability

All preprocessing and analysis scripts are publicly available (https://github.com/AlistairPerry/FTLDMEGMEM). Resources including the unthresholded statistical VBM results, and the anatomical mask region used in atrophy calculation, are also publicly available (https://neurovault.org/collections/12279/).
