# Supplementary material for: The neurophysiological effect of NMDA-R antagonism of frontotemporal lobar degeneration is conditional on individual GABA concentration
Source: Transl Psychiatry. 2022 Aug 27;12:348. doi: 10.1038/s41398-022-02114-6 (PMC9420128; doi:10.1038/s41398-022-02114-6)
Supplement: Supplementary file 1 — Supplementary Material I [file 41398_2022_2114_MOESM1_ESM.pdf]

Supplementary Information:

The neurophysiological effect of NMDA-R antagonism of frontotemporal lobar degeneration is conditional on individual GABA concentration

Alistair Perry[1,2](PhD)†, Laura Hughes[1,2](PhD), Natalie Adams[2](PhD), Michelle Naessens[2](MSc), Alexander G. Murley[2](PhD), Matthew A. Rouse[1](MSc), Duncan Street[2](PhD), P Simon Jones[2](MSc), Thomas E. Cope[1,2](PhD), Ece Kocagoncu[1,2](PhD), James B. Rowe[1,2](PhD)

[1] MRC Cognition and Brain Sciences Unit, University of Cambridge, Cambridge, CB2 7EF, UK

[2] Department of Clinical Neurosciences and Cambridge University Hospitals NHS Trust, University of Cambridge, CB2 0QQ , UK

† Corresponding Author:

Dr. Alistair Perry, Department of Clinical Neurosciences, University of Cambridge, Herchel Smith Building, Forvie Site, Robinson Way, Cambridge Biomedical Campus, Cambridge CB2 0SZ, Email: alistairgperry@gmail.com

1. Supplementary Methods

1.1. *Auditory roving oddball paradigm:*

MEG was recorded while participants completed an auditory roving oddball paradigm [1,2]. Binaural sinusoidal tones (60dB above the population average threshold) were presented for 75ms to participants through headphones, with a 7.5ms ramp up and down at the start and end of the tone, and with stimulus onset asynchrony of 500ms (ITI). This roving oddball paradigm comprises mini-blocks of 3-10 tone repetitions, where at the end of each mini-block the tone frequency changed pseudorandomly. This first tone of each block changing in frequency is defined as the deviant (*dev*) tone. Tone frequencies presented were in the range of 400-800Hz. Participants were under continuous video monitoring to ensure none fell asleep, and they were not asked to attend to the auditory stimuli. The paradigm was performed eyes-open in blocks of five minutes while participants watched a movie (i.e. walking with dinosaurs), with an average number of 1577 (SD=109) stimulus trials (before trial rejection) across each subject and session.

1.2. *MEG data acquisition and preprocessing:*

MEG data were acquired in a magnetically-shielded (IMEDCO) room using the Elekta VectorView system (Elekta Neuromag, Helsinki). This MEG system comprises of 306-channel recordings at 102 spatial locations (a pair of planar gradiometers and a magnetometer at each site) and was sampled at 1000 Hz, with a high-pass filter of 0.03 Hz. Electroocculograms (EOGs) tracked eye movements vertically and horizontally and 5 head position indicator coils tracked head position every 200ms. A 70 channel, MEG-compatible, electroencephalogram (EEG) cap (Easycap GmbH) using Ag/AgCl electrodes positioned according to the 10-20 system was used concurrently, although this modality was not used for rejecting bad trials, source reconstruction, nor in subsequent analysis. Scalp shape was recorded with a 3D digitizer (Fastrak Polhemus Inc., Colchester, VA) using > 100 scalp points, as well as the position of the nasion and bilateral pre-auricular fiducial points. Auditory stimuli were delivered binaurally through MEG-compatible ER3A insert earphones (Etymotic Research). Instructions and the video were presented on the screen positioned 1.22 meters in front of the participant's visual field.

To ensure that the earphones were working correctly, before the MEG recording participants performed an automated hearing test in the scanner. They were presented tones at 1000Hz to either ear with varying loudness, and instructed to press the button when they heard the tone.

Preprocessing of MEG data was performed in SPM12 (www.fil.ion.ucl.ac.uk/spm, v7771), FieldTrip [3] and OSL (https://github.com/OHBA-analysis/osl-core) software packages in Matlab (2019a) (pipeline available at https://github.com/AlistairPerry/FTLDMEGMEM).

First, the raw E/MEG were preprocessed using MaxFilter 2.2.12 in Matlab 2018a (Elekta Neuromag, https://imaging.mrc-cbu.cam.ac.uk/meg/Maxfilter_V2.2). This included interpolation of bad channels, signal source separation to remove noisy signals from outside the brain, and

head motion correction. Subject data was also transformed into a standard space for the analysis of sensor channels.

Next, the data were downsampled to 500Hz, band-pass filtered (0.1-125 Hz using a fifth-order Butterworth filter) and further notch filtered to remove 50 (45-55Hz) and 100 Hz (95-105Hz) line noise [4]. Artefact rejection (*osl_detect_artefacts)* was first used to remove bad channels, and then independent component analysis (ICA) further removed eye-movement related artefacts. ICA involved: A fast fixed-point algorithm, 800 maximum steps, 60 principal components, symmetric approach, tan-h non-linearity, epsilon of 0.00001, via the *FastICA* package for MATLAB. The independent component time series were correlated with the VEOG and HEOG channel time series. The components that revealed correlations higher than $r = 0.35$ were removed and the data of the remaining independent components were reconstructed. Continuous data were epoched between -100 to 400ms relative to auditory stimulus presentation, and bad trials were identified and removed again using OSL's artefact rejection (using magnetometers and gradiometers). Artifact MEG channels and trials were both removed using *osl_detect_artefacts*, which identifies outliers through a Generalized Extreme Studentized Deviate (ESD) test, at the default significant threshold of $\alpha=0.05$. Lastly, trials were averaged using robust averaging and then again low-pass filter corrected (125 Hz) to remove high-frequency noise induced after averaging.

1.3. Independent control cohort

We used the MEG data from an independent control cohort [2] to (1) corroborate calculation of individual MMN responses based upon the *rep3-dev* waveform, and (2) determine that our reported group differences (to FTLD patients) at the sensor level are robust to the control cohort studied. This control population is previously published, and do not differ in age (p=0.69) to the current control cohort.

The MEG data were acquired, preprocessed, and source localised identically to the steps performed in the current control cohort. The only difference is that while for the current cohort T1-weighted images were acquired at 7T, this independent cohort underwent structural MRI (MPRAGE sequence, TE = 2.9ms, TR = 2000ms, 1.1 mm isotropic voxels) using a different 3T Siemens PRISMA scanner.

1.4 Acquisition parameters of T1-weighted structural imaging scan

The majority of individuals completed a 7T T_1-weighted structural scan on a Siemens TERRA scanner (Siemens Healthineers, Erlangen, Germany) (Nova Medical, Massachusetts, USA). The acquisition parameters of the MP2RAGE sequence included: 0.75mm isotropic voxels, TE=1.99ms, TR=4300ms, resolution = 99ms, bandwidth = 250 Hz/px, voxel size = 0.75 mm^3, field of view = 240 × 240 × 157 mm, acceleration factor (A ≫ P) = 3, flip-angle = 5/6° and inversion times = 840/2370ms).

All structural images were visually inspected for motion and scanning artifacts, as well as segmentation accuracy.

1.5 Acquisition and preprocessing of MR Spectroscopy

Magnetic resonance spectra data were acquired in controls and patients as part of a larger cohort study [5]. MRS data were acquired serially from a region-of-interest in the right inferior frontal gyrus (2 x 2 x 2 cm^3). Voxel selection was performed manually using anatomical landmarks from the T_1-weighted structural scan, with Murley et al. [5] (c.f. Fig. 1) demonstrating the consistency of voxel placement across subjects (Fig. 1B in the current study). A control region in the right primary visual cortex was also included. Spectra were acquired using a short-echo semi-LASER sequence [6,7] (repetition time/echo time = 5000/26 ms, 64 repetitions) and we used the recommended pre-scan protocol of FASTESTMAP shimming [8], semi-LASER water-peak flip angle, and VAPOR water suppression calibration [9].

For every participant, each of the 64 individual spectral transients from each participant were saved separately. Eddy-current effects were corrected for and also frequency and phase shifts using MRspa (Dinesh Deelchand, University of Minnesota, www.cmrr.umn.edu/downloads/mrspa).

Neurochemicals between 0.5 and 4.2ppm, including glutamate and GABA, were quantified using LCModel (Version 6.2-3)[10] with water scaling and a simulated basis set that included experimentally-acquired macromolecule spectra. See Fig.1 from [5] for illustration of the MRS Spectrum (from all participants) and LCModel fit for GABA and Glutamate in the inferior frontal gyrus.

Grey matter volume was used to correct for GABA, and grey and white matter volume for glutamate. A generalized linear model (GLM) removed the effect of age, sex, and partial volume [5]. The model was weighted by Cramér-Rao lower bound values (CRLB, SI Table 2), so that participants with less accurate metabolite estimates were penalised to have less influence on the regression. Non-corrected MRS values are also presented.

1.6 Preprocessing and segmentation of anatomical images

For those participants with MP2RAGE images, we first performed signal bias correction [11] and segmentation of grey matter (GM), white matter (WM), and cerebrospinal fluid (CSF) segments using the standard settings in SPM12 (v7771). Next, we created a study-specific template image with differomorphic registration (DARTEL) [12] of native space grey and white matter images from an equal number of control, bvFTD and PSP individuals. Each participant's native GM image were then normalised to standard space by applying the deformation combined with the affine transformation parameters (i.e. native-space to group average to MNI space) which included modulation in order to preserve local volume. The images were lastly smoothed with a Gaussian kernel at 8 mm full width half maximum (FWHM). The total intracranial volume (TIV) for each participant was calculated using the Tissue Volumes function in SPM12.

1.7 Association between prefrontal cortical atrophy and change in MMN response to drug

Grey matter volume (GMV) in the inferior frontal gyrus was calculated from a right-hemisphere anatomical mask combining Brodmann areas 8, 9 and the frontal operculum (OP8) (https://github.com/inm7/jubrain-anatomy-toolbox, v2d7a002)[13], regions which overlapped with the MMN sphere used in MEG analysis (SI Fig. 9A, and is available at https://neurovault.org/images/776918). GMV was calculated from the total sum of voxel probabilities within the above mask enclosing the individual normalized grey-matter images. The normalization included modulation in order to preserve local volume, but were not smoothed.

A linear model was performed to test for the association between the GMV in the inferior frontal gyrus (IV) with the dependent variable, the change in MMN response to memantine (vs. placebo) in the auditory cortex. Total intracranial volume and age were included as covariates in the linear model. Bayesian linear regression was also conducted.

1.8. Voxel-based morphometry

Voxel-based morphometry (VBM) was used in SPM12 to compare GM volume between controls and patients (bvFTD and PSP combined) and across the diagnostic groups. GM volumes for each diagnostic group were compared with independent two-sample t-tests with age, sex and total intracranial volume used as covariates of no interest [14]. Significant effects were identified using cluster-level statistics ($p<0.05$, family-wise error corrected for multiple comparisons) above a height threshold of $p<0.001$ (uncorrected). Unthresholded statistical maps for each contrast are available at https://neurovault.org/collections/12279.

2. Supplementary Figures

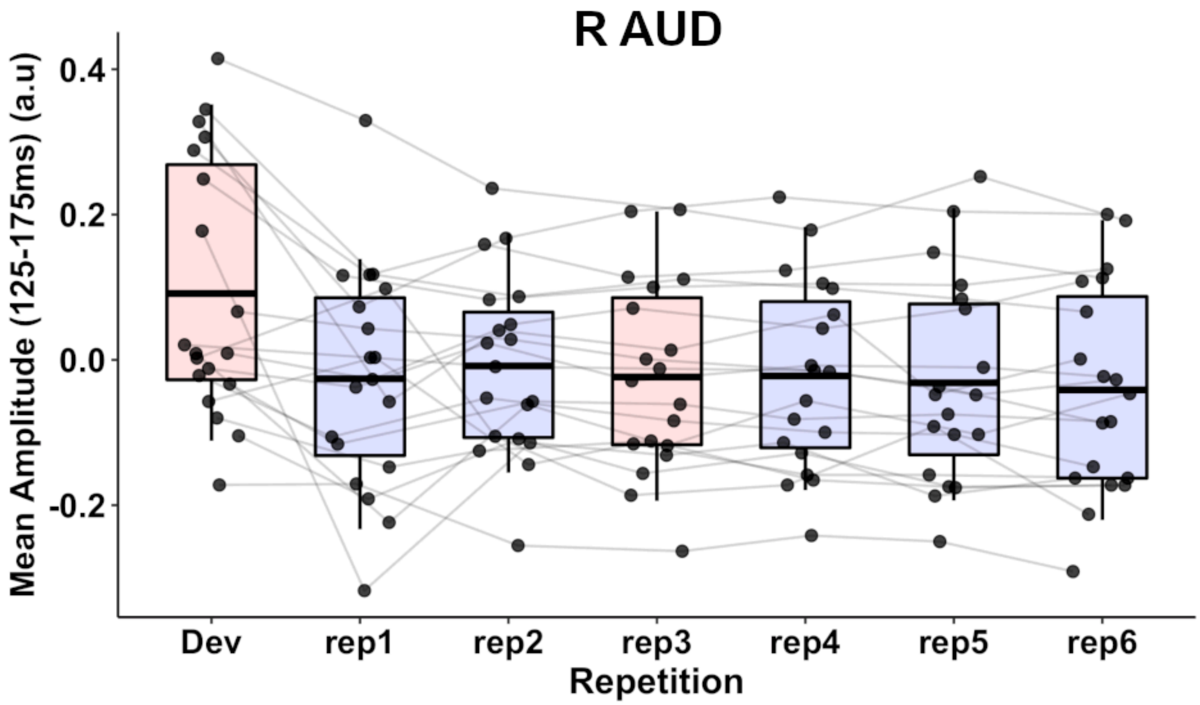

SI Fig. 1 – Repetition-dependent changes in the mean MEG amplitude response to auditory tone presentation within an independent control cohort. Individual data points (black circles and lines) represent changes in each subjects mean MEG amplitude (125-175ms) measured in the right auditory cortex (R AUD), as a function of tone repetition (*dev*, rep_1, …, rep_n) in the oddball paradigm. Boxes represent interquartile range of 25% and 75% percentile, with whiskers indicating 95% probability density.

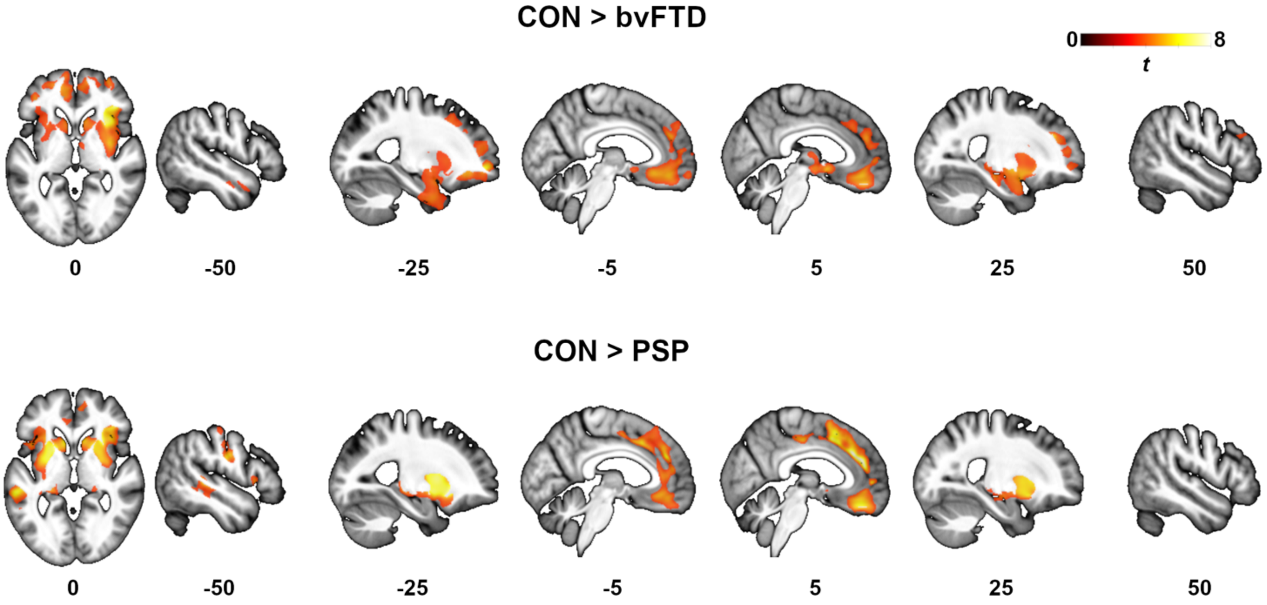

SI Fig. 2 - Neuroanatomical differences across controls and bvFTD and PSP subgroups. (A) Group-wise voxel-based morphometry comparisons (cluster-level, $p < .05$, FWE-corrected; height-threshold, $p < 0.001$, uncorrected). Note, bvFTD and PSP did not differ. Unthresholded SPM maps are available at https://neurovault.org/collections/12279/

bvFTD, behavioral variant Frontotemporal Dementia; PSP, progressive supranuclear palsy

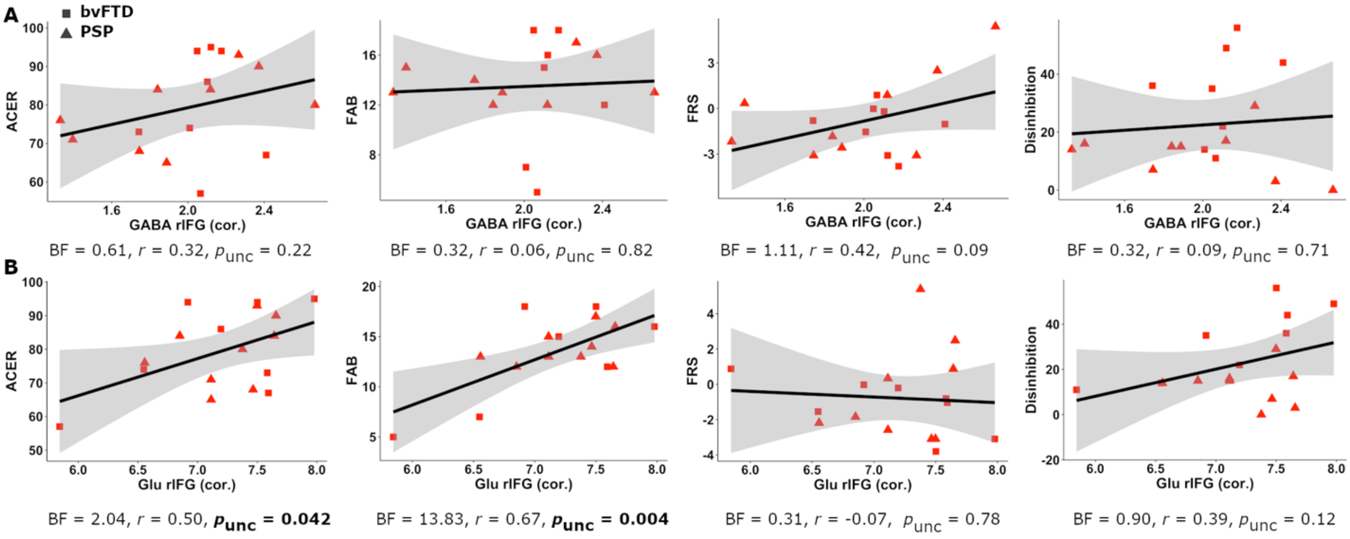

SI Fig. 3 – Association between corrected GABA (A) and Glutamate (B) MRS concentrations in the right inferior frontal gyrus withkey phenotypic markers in persons with bvFTD/PSP. Phenotypic scores include ACER Total Score, Frontal Assessment Battery (FAB), Frontotemporal Dementia Rating Scale (FRS, logit scores) and disinhibition composite (comprised of *abnormal behaviour*, *eating* and *sleep* scales from the CBI-R).

bvFTD, behavioral variant Frontotemporal Dementia; PSP, progressive supranuclear palsy

rIFG, Right Inferior Frontal Gyrus; GABA and Glutamate concentrations corrected for age, sex, and partial volume information

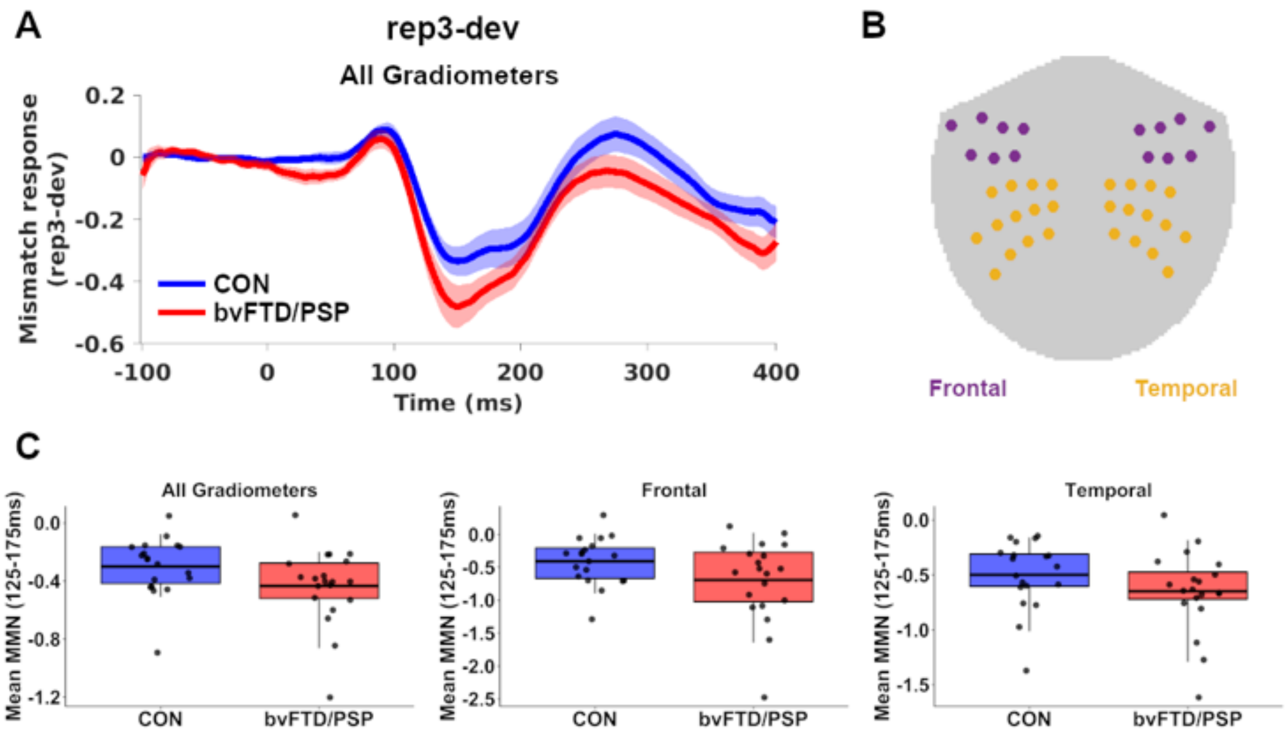

SI Fig. 4– Mismatch responses in MEG sensors across controls and bvFTD/PSP persons on placebo. (A) Group average mismatch responses averaged across all gradiometer sensors for controls (blue line) and bvFTD/PSP (red). Mismatch responses were derived by the difference of the *rep3* and *dev* waveforms, with shading representing standard error at each peri-stimulus time point. (B) Spatial location of MEG gradiometer channels used to measure the average ERF's from representative frontal (purple circles) and temporal (orange) regions. (C) Mean MMN responses (125-175ms) in controls and FTLD across representative sensor spatial locations. Boxes represent interquartile range of 25% and 75% percentile, with whiskers indicating 95% probability density.

bvFTD, behavioral variant Frontotemporal Dementia; PSP, progressive supranuclear palsy

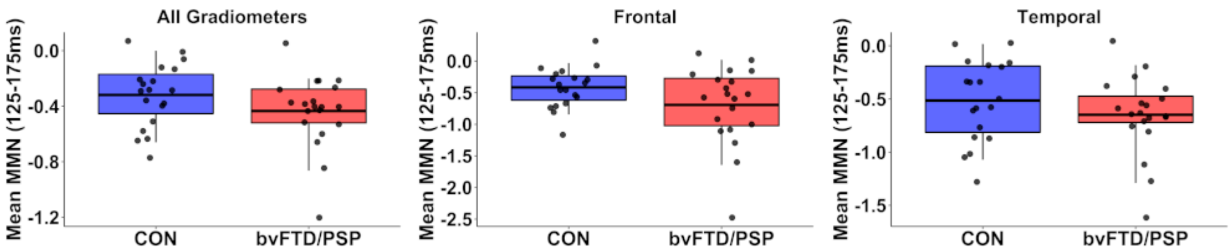

SI Fig. 5 – Mean MMN responses of MEG sensors in bvFTD/PSP persons and an independent control cohort on placebo. Spatial location of frontal and temporal gradiometers are those illustrated in SI Fig.3B. Boxes represent interquartile range of 25% and 75% percentile, with whiskers indicating 95% probability density.

bvFTD, behavioral variant Frontotemporal Dementia; PSP, progressive supranuclear palsy

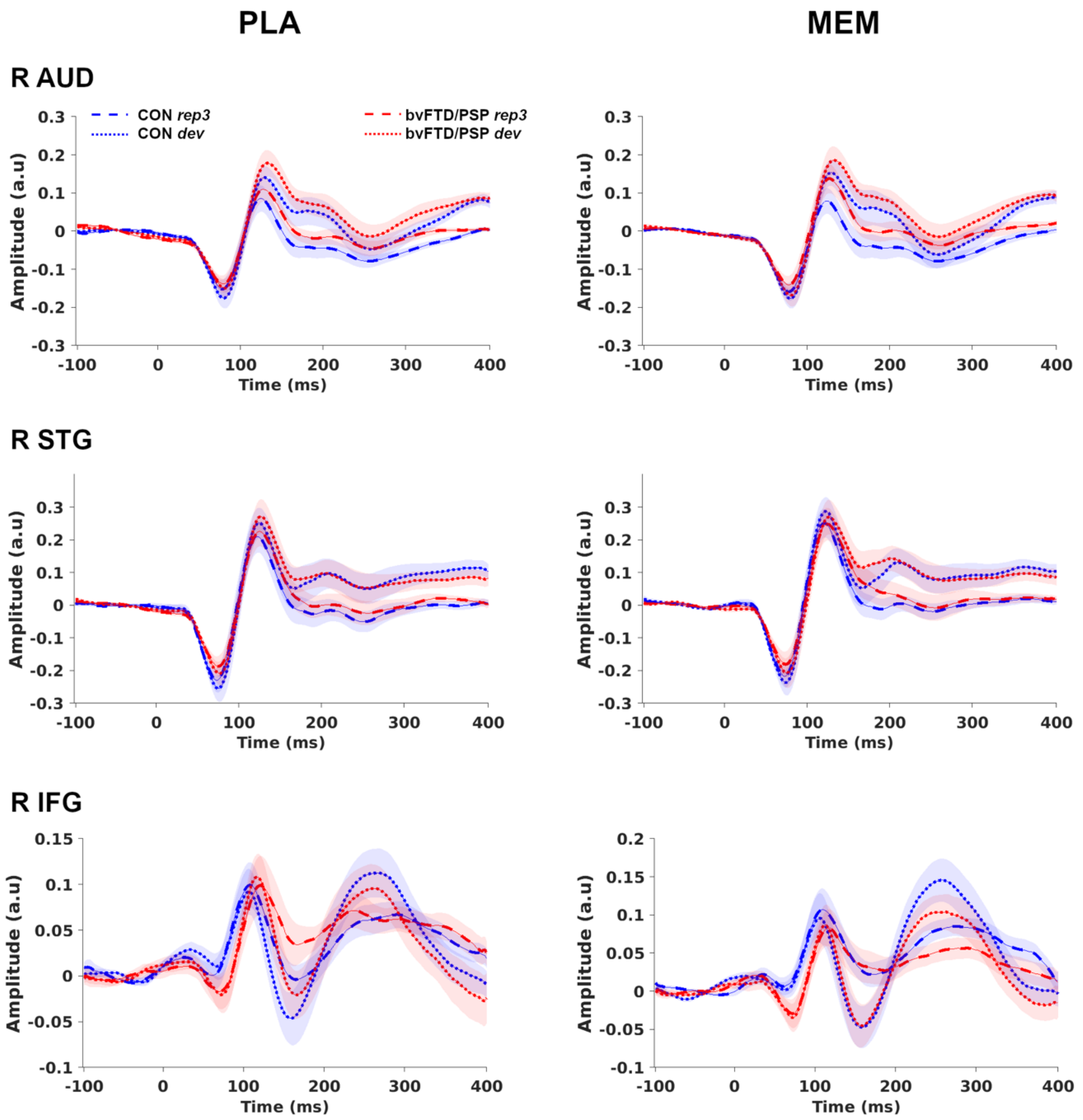

SI Fig. 6 – Group averaged source waveforms of single-condition *dev* and *rep3* responses for controls and bvFTD/PSP persons across placebo (PLA) and memantine (MEM) drug sessions. Dashed and dotted lines represent group average *dev* and *rep3* conditions respectively, with the shading indicating standard error at each time point.

R IFG, Right Inferior Frontal Gyrus; R STG, Right Superior Temporal Gyrus; R AUD, Right Auditory Cortex; bvFTD, behavioral variant Frontotemporal Dementia; PSP, progressive supranuclear palsy

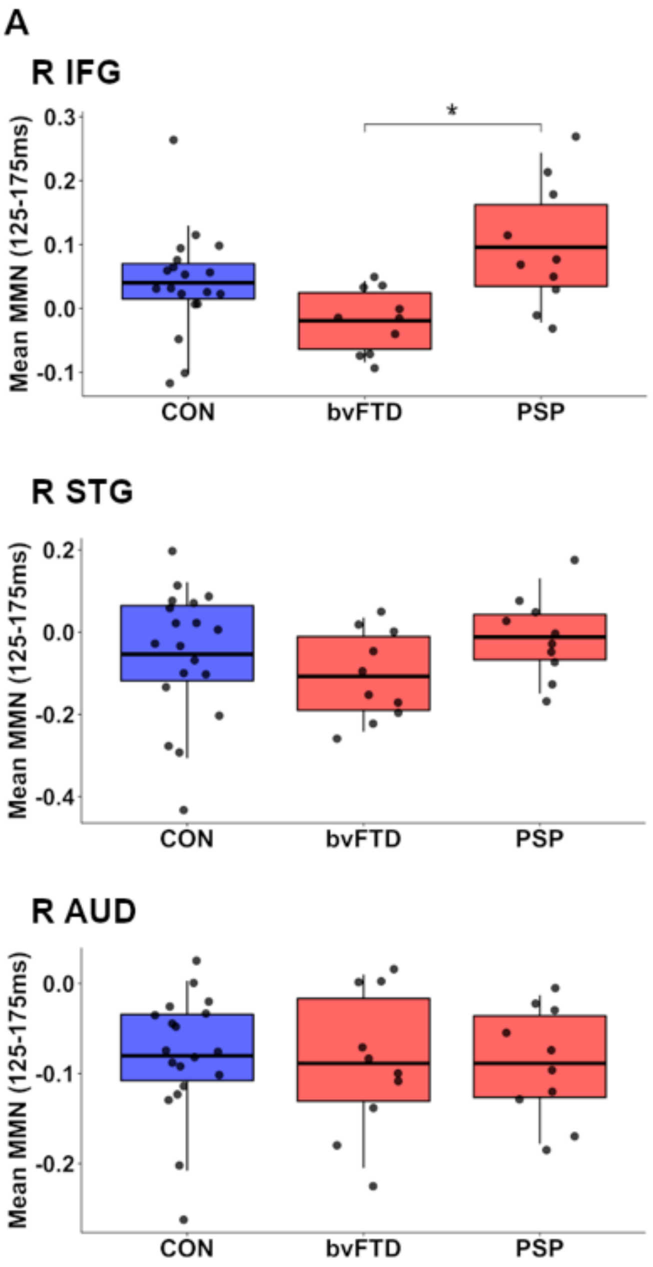

SI Fig. 7 – Auxiliary analyses for mean MMN responses across source regions on placebo in controls and bvFTD/PSP persons. (A) Mean MMN responses across controls (blue) and disease subgroups including bvFTD and PSP persons.

R IFG, Right Inferior Frontal Gyrus; R STG, Right Superior Temporal Gyrus; R AUD, Right Auditory Cortex. bvFTD, behavioral variant Frontotemporal Dementia; PSP, progressive supranuclear palsy

* $p_{tukey} < .05$

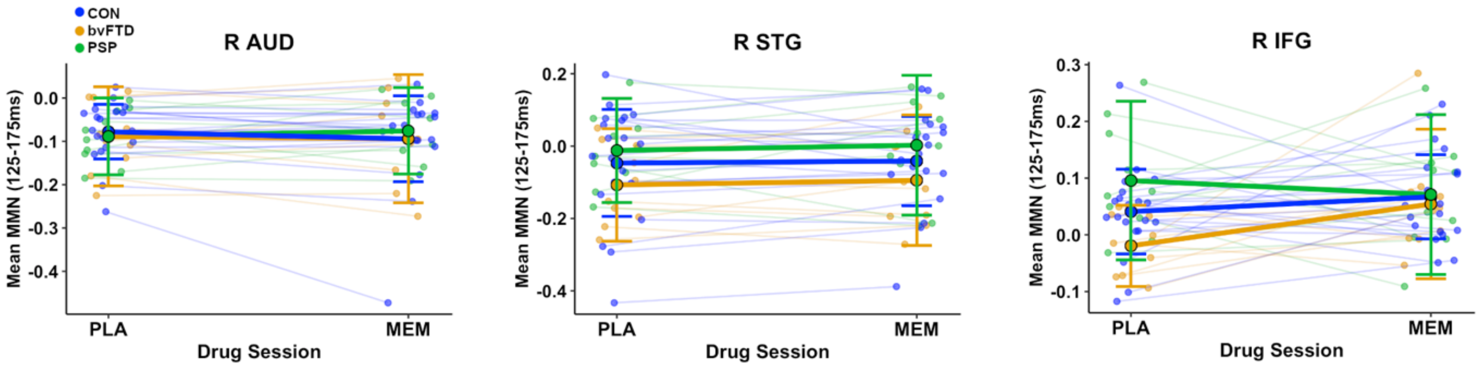

SI Fig. 8 – Auxiliary analysis for group responses to memantine across source regions. Mean MMN responses across controls (blue) and disease subgroups including bvFTD (orange) and PSP (green) persons.

R IFG, Right Inferior Frontal Gyrus; R STG, Right Superior Temporal Gyrus; R AUD, Right Auditory Cortex. bvFTD, behavioral variant Frontotemporal Dementia; PSP, progressive supranuclear palsy

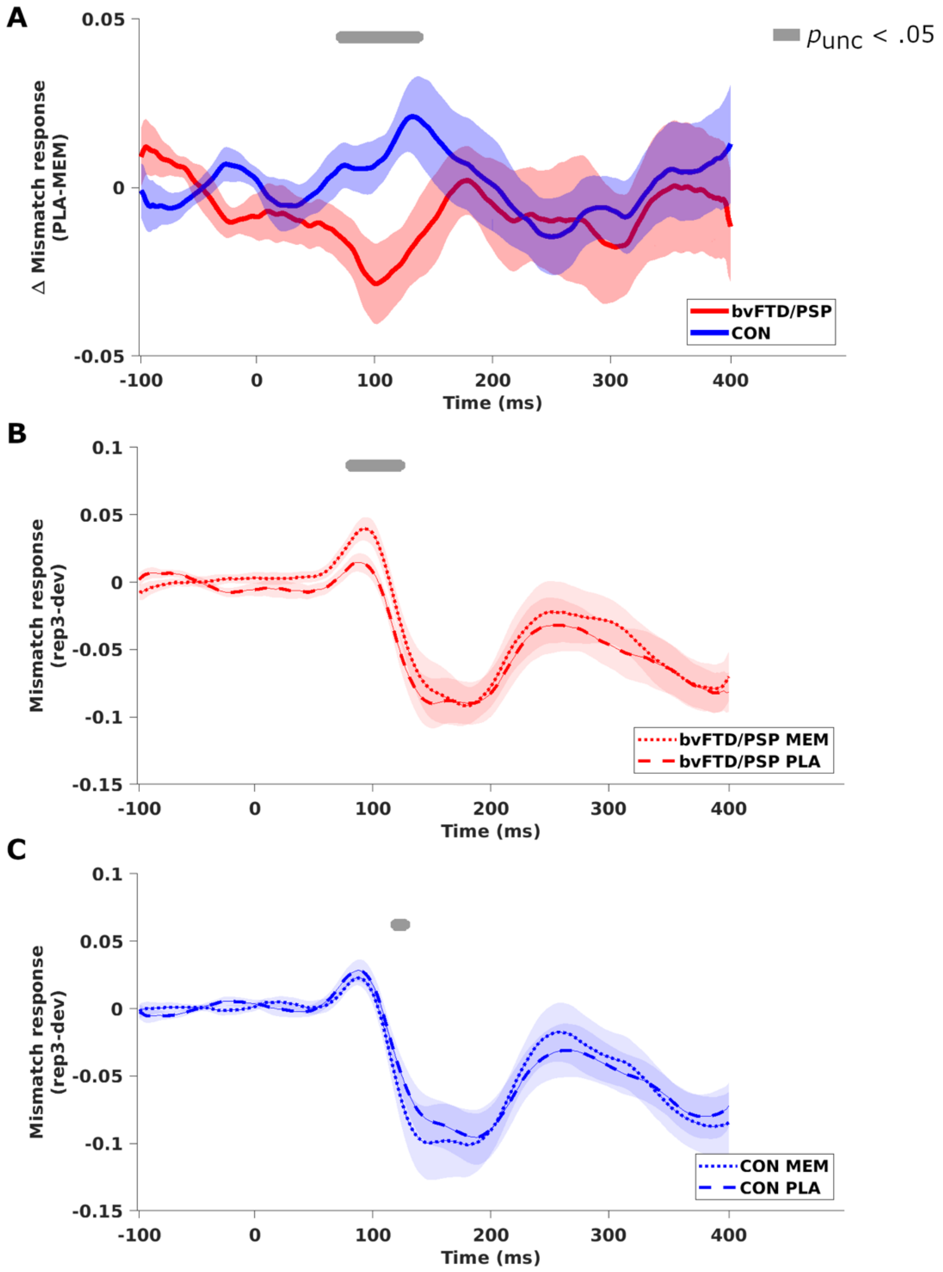

SI Fig. 9 – Auxiliary analysis for group mismatch responses to memantine in the right auditory cortex, across the entire trial window. (A) For control (blue) and bvFTD/PSP groups (red), the average group difference in the mismatch response across memantine (MEM) and placebo (PLA) conditions. Grey markers show time-points indicating (uncorrected) significant group differences (using independent *t*-tests), meaning there is a group difference in the difference of the mismatch responses across drug session. (B) and (C) Mismatch responses as a function of drug session condition, for bvFTD/PSP and control groups respectively. Grey markers show time-points indicating (uncorrected) significant within-group drug effects between the placebo (dashed line) and drug session (dotted). In each panel, shading indicates standard error at each time point.

N.B: These effects do not survive correction for multiple comparisons correction (FDR < 0.05)

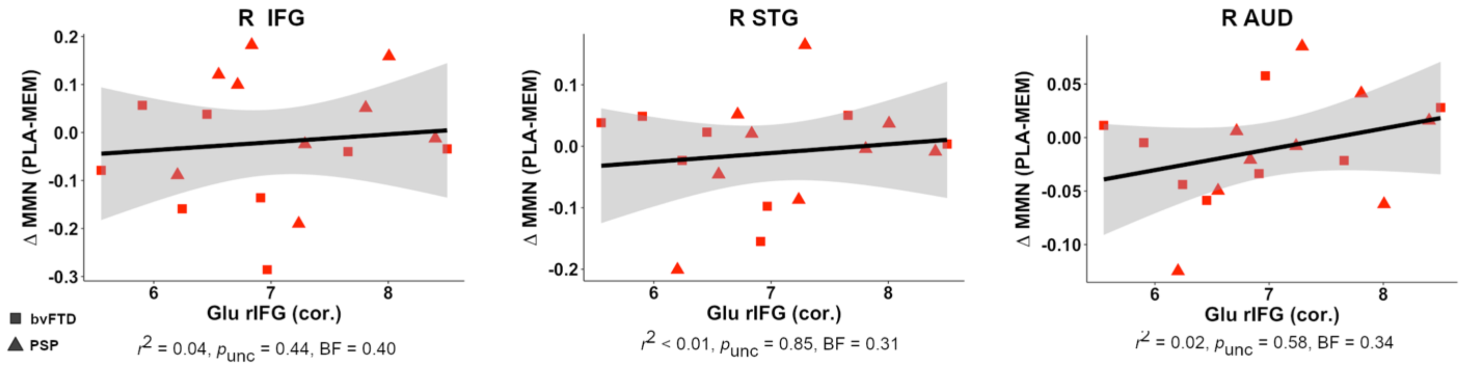

SI Fig. 10 – Influence of baseline MRS glutamate concentration on the response to memantine across source regions. Association between corrected glutamate (Glu) concentrations in the R IFG and change in MMN to memantine (vs. placebo) (PLA-MEM) across bvFTD (squares) and PSP persons (triangles).

R IFG, Right Inferior Frontal Gyrus; R STG, Right Superior Temporal Gyrus; R AUD, Right Auditory Cortex. bvFTD, behavioral variant Frontotemporal Dementia; PSP, progressive supranuclear palsy

Glutamate concentrations corrected for age, sex, and partial volume information

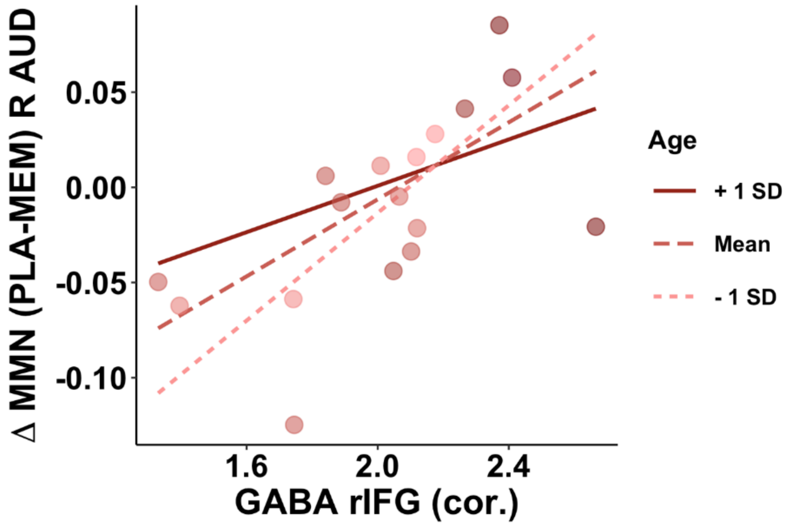

SI Fig. 11 – Association between prefrontal GABA and change in right auditory MMN response to memantine as a function of patients age. Different slopes examine moderation of age on the relationship between MRS concentrations and change in MMN to memantine (compared to placebo) – relatively younger bvFTD/PSP persons (minus 1 standard deviation below mean age) are illustrated in light red, whilst older individuals (1 standard deviation above mean age) are in dark red.

R IFG, Right Inferior Frontal Gyrus

GABA concentrations corrected for age, sex, and partial volume information

3. Supplementary Tables

SI Table 1. Demographic and neuropsychological characteristics of participants by disease subgroup

Attached as separate file

SI Table 2 – MEG trial and spectral quality information in the right inferior frontal gyrus

	Group Means (SD)		Difference	
	**CON**	**bvFTD/PSP**	***p*-val**	**Bayes Factor**
Number MEG trials				
rep3	197 (16.51)	189.45 (9.79)	*	2.87
Dev	214.61 (16.52)	206.5 (10.02)	*	3.99
Bad trials†	24.76 (23.22)	50.90 (68.51)	*	1.95
MRS quality indices				
Line width	13.88 (1.33)	12.66 (2.37)	n.s	0.91
SNR	56.17 (4.02)	46.53 (7.15)	***	94.40
CRLB (Glu)	2 (0)	2.29 (0.47)	n/a	n/a
CRLB (GABA)	9 (0.95)	10.76 (2.73)	*	1.83

*** = $p < 0.001$; ** = $p < 0.01$; * = $p < 0.05$; n.s = non-significant, all uncorrected
† = Number of bad trials (all conditions, i.e. *dev* and rep_1-rep_n) removed in artifact detection
SNR, Signal-to-noise-ratio; CRLB, Cramer-Rao lower bounds; Glu, Glutamate

n/a - variance in controls were equal to zero, violating assumption of equality of variances

SI Table 3 – Voxel based morphometry contrasts. Anatomical and statistical information of significant clusters (cluster-level, p_{FWE}<0.05; peak-level, p<0.001) of grey matter atrophy.

			Cluster-level			Peak-level		
**Contrast**	***df***	***n***	p_{FWE}	k_E	***t***	**x**	**y**	**z**
CON > bvFTD/PSP	1,31	1	< 0.001	198496	7.78	34	0	8
		2	0.003	7968	5.35	52	-4	31
		3	0.019	5191	4.8	-19	-101	0
CON < bvFTD/PSP	-	n.s						
CON > bvFTD	1,23	1	< 0.001	110282	6.93	40	33	1
CON < bvFTD	-	n.s						
CON > PSP	1,22	1	< 0.001	77336	8.29	6	33	31
		2	0.017	4195	6.82	-56	-32	2
		3	0.025	3828	5.56	-51	-10	29
CON < PSP	-	n.s						
bvFTD > PSP	1,13	n.s						
bvFTD < PSP	-	n.s						

N.B: X,Y,Z coordinates refer to local maxima in standard MNI space.

SI Table 4 – Subgroup comparisons of MRS GABA and Glutamate corrected concentrations in the right inferior frontal gyrus

	**CON (n = 12)**	**bvFTD (n = 8)**	**PSP (n = 9)**
Group Means (SD)			
GABA	2.302 (0.34)	2.084 (1.90)	1.958 (0.44)
Glutamate	7.30 (0.42)	7.14 (0.69)	7.25 (0.37)
Group Contrasts			
		GABA	Glutamate
	df	*p*-val (Bayes Factor)	
CON vs bvFTD	18	n.s (0.99)	n.s (0.46)
CON vs PSP	19	n.s (1.52)	n.s (0.40)
bvFTD vs PSP	15	n.s (0.51)	n.s (0.44)

SI Table 5 – ANOVA post-hoc tests for differential subgroup MMN responses in the right inferior frontal gyrus

**Contrast**	***t***	p_{tukey}	**BF_{10}**
CON vs bvFTD	1.90	n.s	1.69
CON vs PSP	-1.79	n.s	0.95
bvFTD vs PSP	-3.22	0.007	10.42

SI Table 6 - Association between patients drug-dependent responses to memantine (relative to placebo) and uncorrected MRS concentrations

	GABA			Glutamate		
**Region**	r^2	p	**BF**	r^2	p	**BF**
R AUD	0.38	**0.017**	4.23	0.10	n.s	0.31
R STG	0.05	n.s	0.43	0.02	n.s	0.34
R IFG	0.33	n.s	0.39	0.01	n.s	0.32

AUD, Auditory Cortex; STG, Superior Temporal Gyrus; IFG, Inferior Frontal Gyrus

References

1 Garrido MI, Friston KJ, Kiebel SJ, Stephan KE, Baldeweg T, Kilner JM. The functional anatomy of the MMN: a DCM study of the roving paradigm. Neuroimage. 2008;42(2):936-44.

2 Adams NE, Hughes LE, Phillips HN, Shaw AD, Murley AG, Nesbitt D, et al. GABA-ergic dynamics in human frontotemporal networks confirmed by pharmaco-magnetoencephalography. Journal of Neuroscience. 2020;40(8):1640-49.

3 Oostenveld R, Fries P, Maris E, Schoffelen J-M. FieldTrip: open source software for advanced analysis of MEG, EEG, and invasive electrophysiological data. Computational intelligence and neuroscience. 2011;2011.

4 Kocagoncu E, Nesbitt D, Emery T, Hughes L, Henson RN, Rowe JB. Neurophysiological and brain structural markers of cognitive frailty differ from Alzheimer's disease. Journal of Neuroscience. 2022.

5 Murley AG, Rouse MA, Jones PS, Ye R, Hezemans FH, O'Callaghan C, et al. GABA and glutamate deficits from frontotemporal lobar degeneration are associated with disinhibition. Brain. 2020;143(11):3449-62.

6 Öz G, Tkáč I. Short-echo, single-shot, full-intensity proton magnetic resonance spectroscopy for neurochemical profiling at 4 T: validation in the cerebellum and brainstem. Magnetic resonance in medicine. 2011;65(4):901-10.

7 Deelchand DK, Adanyeguh IM, Emir UE, Nguyen TM, Valabregue R, Henry PG, et al. Two-site reproducibility of cerebellar and brainstem neurochemical profiles with short-echo, single-voxel MRS at 3T. Magnetic resonance in medicine. 2015;73(5):1718-25.

8 Gruetter R, Tkáč I. Field mapping without reference scan using asymmetric echo-planar techniques. Magnetic Resonance in Medicine: An Official Journal of the International Society for Magnetic Resonance in Medicine. 2000;43(2):319-23.

9 Tkáč I, Starčuk Z, Choi IY, Gruetter R. In vivo 1H NMR spectroscopy of rat brain at 1 ms echo time. Magnetic Resonance in Medicine: An Official Journal of the International Society for Magnetic Resonance in Medicine. 1999;41(4):649-56.

10 Provencher SW. Estimation of metabolite concentrations from localized in vivo proton NMR spectra. Magnetic resonance in medicine. 1993;30(6):672-79.

11 O'Brien KR, Kober T, Hagmann P, Maeder P, Marques J, Lazeyras F, et al. Robust T1-weighted structural brain imaging and morphometry at 7T using MP2RAGE. PloS one. 2014;9(6):e99676.

12 Ashburner J. A fast diffeomorphic image registration algorithm. Neuroimage. 2007;38(1):95-113.

13 Eickhoff SB, Stephan KE, Mohlberg H, Grefkes C, Fink GR, Amunts K, et al. A new SPM toolbox for combining probabilistic cytoarchitectonic maps and functional imaging data. Neuroimage. 2005;25(4):1325-35.

14 Barnes J, Ridgway GR, Bartlett J, Henley SM, Lehmann M, Hobbs N, et al. Head size, age and gender adjustment in MRI studies: a necessary nuisance? Neuroimage. 2010;53(4):1244-55.
